# Preoperative Risk Score for Early Mortality After Up-Front Pancreatic Cancer Surgery: A Nationwide Cohort Study

**DOI:** 10.1007/s00268-022-06678-8

**Published:** 2022-08-08

**Authors:** Paulina Bereza-Carlson, Johan Nilsson, Bodil Andersson

**Affiliations:** 1 Department of Clinical Sciences Lund, Surgery, Lund University, Lund, Sweden; 2 Central Hospital of Kristianstad, Kristianstad, Sweden; 3 Department of Clinical Sciences Lund, Cardiothoracic Surgery, Lund University, Lund, Sweden; 4 Skåne University Hospital, Lund, Sweden

## Abstract

**Background:**

Pancreatic ductal adenocarcinoma is a highly fatal malignancy. The aim was to identify preoperative factors for early mortality in up-front resectable patients following pancreatoduodenectomy (PD) and develop an early mortality risk score.

**Methods:**

Patients registered in the Swedish National Registry for Pancreatic and Periampullary Cancer were included. Relevant preoperative factors (*n* = 21) were investigated. Early mortality was defined as death within 12 months after surgery. Based on the identified risk factor odds ratios (ORs), the Score Predicting Early Mortality (SPEM) was developed.

**Results:**

In total, 2183 PDs were performed, and 926 patients met the study criteria. The mean age was 68 (SD ± 8.8) years, and 48% were female. A total of 233 (24%) patients died within 12 months. In the multivariable analyses, age > 75 years (OR 1.7; 95% CI 1.1–2.4; *p* = 0.008), CRP ≥ 15 mg/L (OR 2.0; 95% CI 1.3–3.1; *p* = 0.001), CA 19-9 > 500 U/mL (OR 1.8; 95% CI 1.0–3.2; *p* = 0.040), diabetes mellitus (OR 1.40; 95% CI 1.00–2.1; *p* = 0.042), and active smoking (OR 1.47; 95%CI 1.00–2.00; *p* = 0.050) were found to be independent risk factors for early mortality.

**Conclusion:**

Five independent preoperative risk factors for early mortality following PD were identified and together formed SPEM. The score might be a useful tool in establishing individualized treatment plans.

## Introduction

Pancreatic cancer is the third leading cause of cancer death in Europe and the USA [1], and it is expected to become the second by 2030 [2].

The majority of patients with pancreatic ductal adenocarcinoma (PDAC) first show symptoms when the disease is already locally advanced and/or metastasized, and only approximately 20–25% of patients are potentially eligible for surgery [3].

Adequate diagnosis and treatment of PDAC represent a great challenge. The identification of early biomarkers can hopefully improve the diagnosis and treatment of patients with PDAC in the future [4]. Until identification is feasible, it is crucial to carefully select patients for surgery.

Among all patients eligible for upfront surgery, those who survive only a short time (< 12 months) should ideally be identified preoperatively. Upfront surgery may not be appropriate for these patients, and it may be worthwhile to consider alternative treatment options such as neoadjuvant chemotherapy [5, 6]. Identification of patients at high risk of early mortality might therefore be useful in individualized treatment strategies. This in turn would potentially avoid subjecting unsuitable candidates to surgery and preserve better quality of life for this group of patients.

The available literature describes predominant risk factors for short-term survival related to tumor characteristics, including tumor size and lymph node involvement [7, 8]. Favorable prognosis is known for curative resection (no residual tumor or R0) and absence of lymph node metastases [9–11]. Preoperative serum values of CA 19-9 have been reported to be associated with margin and/or lymph node status and thus can have predictive value for early recurrence [12–15].

We conducted a nationwide, multicenter cohort study. The primary aim was to identify preoperative prognostic factors predicting early mortality (< 12 months) in patients subjected to upfront pancreatoduodenectomy due to PDAC, and the secondary aim was to create a risk score model to stratify patients’ risk for early mortality.

## Method

This study is based on the Swedish National Registry for Pancreatic and Periampullary Cancer, which contains data on patients with pancreatic or periampullary cancer, as well as all patients undergoing pancreatic surgery. It represents a multicenter, nationwide, nonselected cohort. The registry started in January 2010 and has a high rate of coverage (over 90%, as compared to the Swedish Cancer Registry). The registry was validated in 2016 and 2019 [3].

Patients who underwent PD in Sweden from January 2010 through October 2017 were selected and analyzed. The criteria for inclusion were patients who underwent PD with pathologically confirmed PDAC and a follow-up of at least 12 months after surgery (or death before 12 months). Patients who received neoadjuvant chemotherapy before surgical resection were excluded from the study. This guarantees a homogenous population of primary up-front resectable patients. Early mortality was defined as death before 12 months from the date of surgical resection. The study cohort was divided into two subcohorts: the S-group with patients with a short postoperative survival time (less than 12 months) and the L-group with long-term survivors (defined as at least 12 months).

The registry includes demographic, clinical, and histopathological data as well as information regarding treatment and complications during and after treatment. Preoperative staging is based on computed tomography (CT) of thorax and abdomen. In case of unclear findings, additional imaging techniques are used: endoscopic ultrasound (EUS), magnetic resonance imaging (MRI), or contrast-enhanced transabdominal ultrasound (CEUS). Based on available data and clinical relevance, the following factors were analyzed: age, sex, body mass index (BMI), tobacco use, blood pressure, heart rate, electrocardiogram (ECG) changes, American Society of Anesthesiologists (ASA) score, World Health Organization (WHO) performance status, involuntary weight loss, cardiovascular comorbidity, diabetes mellitus (DM), preoperative biliary drainage, pathologic descriptors (type of tumor, vascular involvement), blood test markers (carbohydrate antigen (CA) 19-9, serum hemoglobin (Hb), C-reactive protein (CRP), white blood cell count (WBC), serum bilirubin level), and waiting time from diagnosis to surgical treatment.

To evaluate the influence of age on mortality after PD, we categorized patients into two age groups: ≤ 75 and > 75 years of age [16, 17]. Cardiovascular comorbidity according to the registry was defined as one of the following: use of at least one of the following drugs such as diuretics, digoxin, antihypertensive medications, warfarin; peripheral edema; cardiomegaly or increased jugular pressure. Not having any of the above mentioned factors is defined as no cardiovascular comorbidity. Deviating ECG means atrial fibrillation and other arrhythmias, including more than 5 extrasystoles per minute, as well as Q or ST-T abnormalities. Systolic blood pressure was registered as intervals: ≤ 89 mmHg, 90–99 mmHg, 100–109 mmHg, 110–130 mmHg, 131–170 mmHg, or ≥ 171 mmHg. The normal range for systolic blood pressure was established as 100–130 mmHg. All values below and above this range were defined as abnormal blood pressure. Normal heart rate was defined as 50–80 beats per minute. All deviations from this norm are defined as abnormal heart rate. BMI was expressed according to the WHO definition, with overweight defined as BMI ≥ 25 [18]. To estimate inflammatory status, analysis of CRP and WBC was performed. Elevated CRP levels were defined as > 3 mg/dL, and elevated WBC counts were defined as ≥ 10 × 10^9^/L. To assess the influence of increasing CRP on mortality, an analysis of CRP ≥ 15 mg/dL was performed. A cutoff of 15 corresponds to approximately the upper quartile of CRP levels in the study cohort. Anemia was measured by Hb. Based on the WHO definition of anemia (adult nonpregnant women < 120 g/L, adult men < 130 g/L), a cutoff at Hb < 120 g/L was established for the study cohort without taking into account the sex. Given the value of the upper quartile of serum bilirubin level in the examined population, a cutoff of more than 45 µmol/L was determined as a potential risk factor for early mortality. Elevated CA 19-9 was defined as > 35 U/mL according to standard laboratory cutoff, and highly elevated CA 19-9 was defined as > 500 U/mL (upper quartile for study cohort 439 U/mL) [12–15]. Preoperative assessment of pathological descriptors (type of tumor and vascular involvement) was based on imaging techniques: CT scans and if needed EUS and MRI. Tumor types are defined as solid, cystic, IPMN or undefined in the registry. Vascular involvement is defined as any arterial tumor invasion (superior mesenteric artery, common hepatic artery, celiac axis) or venous tumor invasion (superior mesenteric vein, main portal vein, or both). Radiological signs of vascular involvement (contact with tumor, deformity, vessel narrowing, occlusion) were assessed according to NCCN Clinical Practice Guidelines [19].

### Statistics

Continuous variables are presented as medians (and interquartile ranges) or means (± SD), and categorical variables are presented as absolute numbers and percentages. Baseline characteristics between groups were compared using the Mann-Whitney *U* test for continuous variables and the chi-square test for categorical variables. Two-sided *p*-values were computed, and a difference was considered statistically significant at a *p* value < 0.05. Univariable and multivariable logistic regression analyses were used to investigate predictors of death 12 months after surgery. Predictors of early death with *p* < 0.25 in univariable analysis and clinical relevance were entered into multiple logistic regression. Patients with early mortality and patients for whom follow-up data were available were included in the analysis. An additive scoring model, Score Predicting Early Mortality (SPEM), was developed based on the odds ratio (OR) for the identified independent risk factors. Missing data were handled with a multiple imputation techniques [20]. Kaplan–Meier estimates of the survivor function were used to estimate long-term survival. The log-rank test was used to compare survival differences between the groups.

Statistical analysis was performed, and graphs were produced using Stata MP statistical package version 15.1, 2017 (StataCorp LP, College Station, Texas, USA).

### Ethics

The study protocol was approved by the Regional Human Ethics Committee at Lund University, Dnr 2015/393.

## Results

### Baseline characteristics of study cohort

In total, 3994 patients with pancreatic tumors were identified during the study period. Among them, 2183 underwent surgical resection with PD and 926 patients with histopathologically confirmed PDAC were finally included. PDAC was pathologically confirmed in 927 patients with no preoperative neoadjuvant treatment and at least 12 months of follow-up or registered early death. Patients with other diagnoses (*n* = 930), neoadjuvant-treated PDAC patients (*n* = 31), PDAC patients with follow-up less than 12 months (*n* = 30) and missing in the histopathology result or follow-up data (*n* = 266) were excluded. Furthermore, one patient was excluded from the study cohort due to an incorrect date of surgical resection. Finally, the study cohort consisted of 926 patients who underwent upfront PD due to PDAC.

The mean age of the patients was 68.0 ± 8.8 years, and 48.3% were female. Patients of older age (> 75 years) constituted 24.4% of all patients (*n* = 225). Mean BMI was 25.2 kg/m^2^. Overweight classification (BMI ≥ 25) was noted in 46.1% of patients (*n* = 410), and 12.7% of patients (*n* = 113) were obese (BMI ≥ 30). Approximately three-quarters of patients (76.8%) met the ASA 1 or 2 criteria, which means that 23.8% of the examined population had at least one severe systemic disease. Baseline characteristics are provided in Table 1.

**Table 1:** Preoperative characteristic of the Study Population with comparison of variables between the long-term (L-group) and short-term (S-group) survivors after surgical resection for PDAC, univariable analysis

Variables	*N*	Study population totally	L-group *N* = 693	S-group *N* = 233	*p*-value
Age*	924	68.0 (± 8.8)	67.34 (± 9.0)	69.9 (± 7.9)	** < 0.001**
Female gender	926	447 (48.3%)	344 (49.6%)	103 (44.2%)	0.151
BMI*	889	25.2 (± 4.5)	25.27 (± 4.4)	25.0 (± 4.7)	0.204
ASA > 2	926	220 (23.8%)	114 (20.8%)	76 (32.6%)	** < 0.001**
WHO performance status	926	109 (11.8%)	101 (14.6%)	43 (18.5%)	0.893
Involuntary weight loss	913	539 (59.0%)	399 (58.3%)	140 (61.1%)	0.455
Active smoking	900	161 (17.9%)	111 (16.4%)	50 (22.2%)	**0.050**
Diabetes mellitus	924	203 (22.0%)	138 (19.9%)	65 (28.0%)	**0.010**
Heart disease	926	313 (33.8%)	224 (32.3%)	89 (38.2%)	0.101
Deviating ECG	926	144 (15.6%)	101 (14.6%)	43 (18.5%)	0.157
Abnormal blood pressure	901	394 (43.7%)	297 (44.1%)	97 (42.5%)	0.676
Abnormal pulse	892	875 (98.1%)	651 (97.9%)	224 (98.7%)	0.456
P- Haemoglobin (g/L)*	900	128.1 (± 14.3)	128.30 (± 14.2)	127.5 (± 14.7)	0.410
P- WBC* (*10^9^/L)	877	8.2 (± 5.3)	8.21 (± 6.0)	8.2 (± 2.5)	0.116
P- CRP (mg/L)*	857	15.2 (± 27.4)	14.22 (± 26.8)	18.0 (± 28.9)	**< 0.001**
P- Bilirubin (µmol/L)*	889	43.8 (± 62.0)	41.91 (± 58.6)	49.3 (± 70.9)	0.142
Biliary drainage before surgery	922	720 (78.1%)	537 (77.8%)	183 (78.9%)	0.737
CA 19-9 (U/mL)*	651	2499.7 (± 23,341.8)	1769.5 (± 13,886.8)	4759.3 (± 40,439.6)	**< 0.001**
Solid tumor type#	926	104 (11.2%)	84 (12.1%)	20 (8.6%)	0.139
Vascular involvement	912	163 (17.9%)	113 (16.6%)	50 (21.6%)	0.090
Time from diagnosis to surgery*	915	29.7 (± 27.6)	28.9 (± 21.6)	32.2 (± 40.3)	0.478

Only patients who died within 12 months or followed up with 12 months are included

Data presented for categorical variables as absolute numbers (percentage) and for continuous variables* as mean (standard deviation)

*BMI* Body Mass Index; *ASA* American Society of Anesthesiologists; *WHO* World Health Organization; *CRP* C-reactive protein; *WBC *White Blood Count; *CA19-9*, Carbohydrate Antigen 19-9

*p* values for continuous variables* Mann–Whitney *U-*test and for discrete variables Chi-squared

#Based on results from preoperative imaging techniques

Bold values indicate statistical significance *p* < 0.05

### Survival outcomes and groups analysis

For the entire group, 30- and 90-day survival rates were 97.8% (906/926) and 96.6% (895/926), respectively. In total, 233 (25.2%, 233/926) patients died within 12 months (S-group).

When comparing the S- and L- groups, mortality before 12 months was statistically significantly associated with older age, ASA scores greater than 2, higher CA 19-9 and CRP levels, the presence of diabetes mellitus, and active smoking. Tumor vascular involvement was more common in the S-group, although it did not reach a significant level. Factors such as BMI, preoperative bilirubin levels, and preoperative biliary tract drainage did not differ between the groups. Detailed results are presented in Table 1.

Multivariable logistic regression analysis included all predictors from the univariable analysis with *p* < 0.25 and clinically relevant variables (Table 2). The ASA score was not included because it is a subjective factor, and it correlated with other analyzed predictive factors.

**Table 2:** Univariable and multivariable analysis identifying predictors for death before 12 months after pancreatoduodenectomy

	Event/total	Univariable analysis			Multivariable analysis		
		OR	95% CI	*p*-value	OR	95% CI	*p*-value
Age > 75 years	73/225	1.58	1.11–2.25	0.011	1.7	1.1–2.4	0.008
CRP ≤ 3 mg/L	45/274	1			1		
> 3–15 mg/L	109/371	2.1	1.4–3.1	< 0.001	1.9	1.3–2.8	0.001
≥ 15 mg/L	65/212	2.3	1.5–3.5	< 0.001	2.0	1.3–3.1	0.001
CA 19-9 ≤ 35 U/ml	28/162	1			1		
36–500 U/ml	76/319	1.5	0.9–2.4	0.101	1.3	0.8–2.1	0.323
> 500 U/ml	55/170	2.3	1.4–3.8	0.002	1.8	1.0–3.2	0.040
Diabetes mellitus	65/203	2.59	1.50–4.46	0.001	1.40	1.0–2.1	0.042
Active smoker	50/161	1.85	1.34–2.55	< 0.001	1.47	1.0–2.0	0.050
Female gender	103/447	0.8	0.6–1.1	0.151			
BMI < 18.5	12/36	1					
18.5–24.9	117/443	0.7	0.3–1.5	0.370			
≥ 25	92/410	0.6	0.3–1.2	0.142			
Days to surgery		1.0	1.0–1.0	0.132			
Solid tumor	20/104	0.7	0.4–1.1	0.141			
Vessel involvement	50/163	1.4	0.9–2.0	0.091			
Heart disease	89/313	1.3	0.9–1.8	0.101			

Clinically relevant variables with *p* < 0.250 in the univariable analysis were included in the multivariable analysis

*OR* Odds ratio; *CI* Confidence interval; *CRP* C-reactive protein mg/L; *CA 19-9* Carbohydrate antigen 19-9; *BMI* Body Mass Index; *Hb* Hemoglobin

**Fig. 1:**
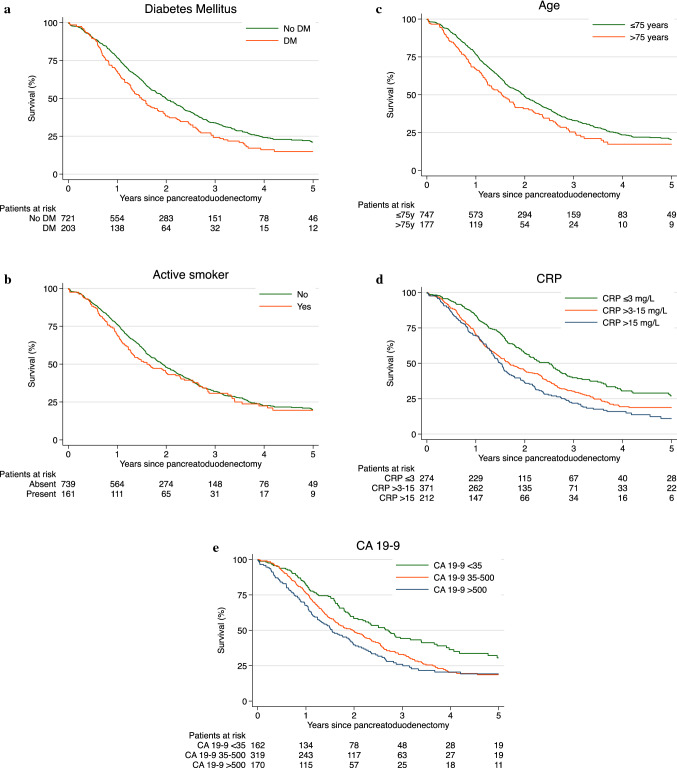
Risk factors for death within 1 year after PD (correlation between survival and time after PD): **a** diabetes, **b** active smoking, **c** age, **d** CRP, **e** CA19-9

In the final multivariable model, age > 75 years, active smoking, diabetes mellitus, elevated CRP ≥ 15 mg/L, and CA 19-9 > 500 U/ml were confirmed as independent risk factors for early mortality after upfront surgery due to PDAC (Table 2). Survival curves are presented in Fig. 1a–e.

### Evaluation of the Score Predicting Early Mortality (SPEM)

**Fig. 2:**
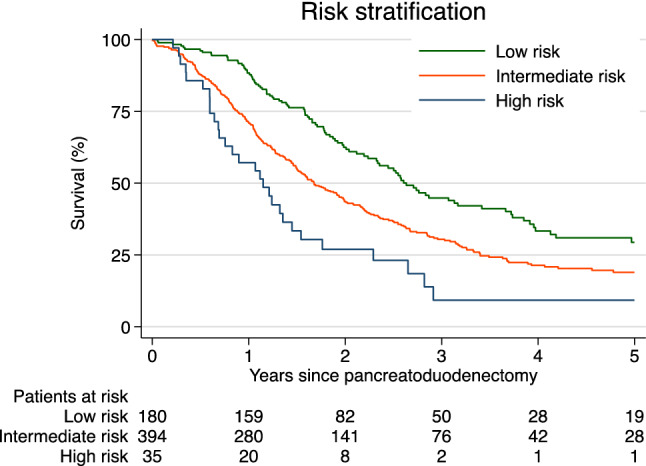
Overall survival, by preoperative predictive score SPEM, Log Rank test

To stratify risk for early mortality after PD, a preoperative predictive scoring was created based on preoperative risk factors identified in the multivariable logistic regression analysis. Scoring distribution was established based on the OR for each risk factor. As a result, elderly patients > 75 years of age (OR 1.7, *p* = 0.008), patients with elevated CA-19-9 > 500 U/ml (OR 1.8, *p* = 0.040), patients with diabetes mellitus (OR 1.40, *p* = 0.042), and active smokers (OR 1.47, *p* = 0.050) add 1.5 points each. Elevated CRP ≥ 15 mg/L (OR 2.0, *p* = 0.001) adds 2 points. The maximal possible score is 8.0 points. Considering the number of received points, patients were stratified into three risk groups for early death after upfront surgery for PDAC: low (0 points), intermediate (1.5–3.5 points), and high risk (> 3.5 points). The distribution of overall survival time according to the preoperative predictive score is presented in Fig. 2. Only patients without missing values in the five risk factors were included.

## Discussion

By analyzing data from the Swedish National Registry for Pancreatic and Periampullary Cancer we identified five preoperative risk factors, including age, active smoking, diabetes mellitus, CRP, and CA 19-9, for early mortality after pancreatoduodenectomy for patients with histopathologically proven PDAC. Based on the odds ratio for each independent risk factor, an early mortality risk score was created.

Steadily aging populations and thus increasing gastrointestinal cancer prevalence have led to a growing interest in the effect of advanced age on survival after major surgery. Elderly cancer patients represent a heterogeneous group, and functional status is the limiting factor of therapeutic options, more than chronological age alone [17, 21, 22]. Results of the present study are in line with findings from other studies [23, 24]. Sho et al. [25] suggest that postoperative prognosis in octogenarians is worse than that in younger patients due to less frequent completion of adjuvant therapy. In contrast, an extensive meta-analysis provided by Pędziwiatr et al.[26] showed that older patients had a higher risk of death and complication rates in the form of delayed gastric emptying (DGE), surgical site infections (SSIs), and pulmonary and cardiovascular complications. It is indisputable that with increasing age, functional reserves of organs and systems decrease significantly, making elderly patients more sensitive to surgical interventions [27].

Increased plasma CA 19-9 levels are well described as both a diagnostic and a prognostic tool for PDAC [12–15, 28]. Hartwig et al. [13] evaluated the correlation of preoperative CA 19-9 levels with resectability, stage of disease, and patient survival. CA 19-9 was significantly higher in patients with progressed disease than in early-stage tumors.

The prognostic value of DM regarding survival after pancreatic cancer surgery is controversial. Many studies identified reduced survival of diabetic patients with PDAC [29–32]; however, there are also studies that do not support a negative effect of DM on survival [33]. A recent meta-analysis was implemented by Lv et al. [34] and presented a negative impact of DM on survival after surgical treatment of pancreatic cancer.

Smoking is an established risk factor for developing PDAC in the general population [35]. Surprisingly few studies have addressed the association of smoking with survival after pancreatic cancer diagnosis. Recently published meta-analysis by Ben et al. [36] shows that both current and former smokers had an elevated risk of mortality when diagnosed with pancreatic cancer.

CRP has been demonstrated to be inversely proportional to survival in a number of malignancies, including pancreatic cancer [15, 37]. Stevens et al.[38] performed a meta-analysis of ten original studies reporting outcomes after pancreatic resection in patients with high CRP, high neutrophil-lymphocyte ratio (NLR), or both. Low CRP was found in nine of ten studies as an independent factor associated with longer survival.

Upfront resection followed by adjuvant chemotherapy is a universally well-accepted standard of treatment for primary resectable pancreatic cancer [39, 40]. Completion of multimodal treatment is an ideal goal. Patients with borderline and locally advanced diseases can receive neoadjuvant chemotherapy as downstaging before surgical resection [41]. Extensive preoperative diagnostic defines staging of the tumor and assessment of respectability. SPEM can become a useful additional tool to ordinary preoperative staging algorithms aiming to identify primary resectable patients at risk for a dismal survival. Prospective validation of SPEM is needed before clinical use. Neoadjuvant chemotherapy followed by surgery can be an alternative treatment protocol for these patients. Alternate sequences of treatment with neoadjuvant chemotherapy instead of upfront surgery could potentially give many benefits, including no delay for systemic treatment. At the same time, identifying aggressive, progressive, or occult metastatic PDAC prior to surgery would decrease mortality after PD and probably preserve better QoL for high-risk patients. To date, several randomized clinical trials (RCTs) with small sample sizes have reported benefits and significantly longer survival for patients with resectable or borderline PDAC who underwent neoadjuvant chemotherapy instead of upfront surgery [5, 6]. Large, well-designed, multicenter phase III RCTs, such as Nordic NorPACT-1 and German NEOPAC, are ongoing and aim to determine the additional benefit of neoadjuvant chemotherapy compared to standard treatment only [42–44].

Previous studies trying to establish preoperative risk factors have different limitations, including the size and diversity of the studied cohort and the type of analyzed variables [45–49].

The strength of our study is the inclusion of all patients in a nationwide, nonselected large cohort. Moreover, all the included patients with PDAC were pathologically confirmed. Additionally, the results of our study can be interpreted as representative of the entire population with primary resectable PDAC.

The current study has limitations inherent to the analysis of registries, e.g., the availability of specific data, the quality of the source data, and the amount of missing data [48]. Due to low coverage of oncological part of registry the influence of adjuvant treatment on the outcome could not be analyzed.

## Conclusion

The current study identified five preoperative risk factors for mortality during the first year after pancreatic cancer surgery and introduced a new risk score, SPEM. Better preoperative risk stratification is one important piece of the puzzle for improved and individualized patient care.
